# Computational Surprisal Analysis Speeds-Up Genomic Characterization of Cancer Processes

**DOI:** 10.1371/journal.pone.0108549

**Published:** 2014-11-18

**Authors:** Nataly Kravchenko-Balasha, Simcha Simon, R. D. Levine, F. Remacle, Iaakov Exman

**Affiliations:** 1 NanoSystems Biology Cancer Center, Division of Chemistry, Caltech, Pasadena, California, United States of America; 2 Software Engineering Department, The Jerusalem College of Engineering, Azrieli, Jerusalem, Israel; 3 The Institute of Chemistry, The Hebrew University, Jerusalem, Israel; 4 Department of Molecular and Medical Pharmacology, David Geffen School of Medicine, University of California Los Angeles, Los Angeles, California, United States of America; 5 Département de Chimie, Université de Liège, Liège, Belgium; Universidad de Granada, Spain

## Abstract

Surprisal analysis is increasingly being applied for the examination of transcription levels in cellular processes, towards revealing inner network structures and predicting response. But to achieve its full potential, surprisal analysis should be integrated into a wider range computational tool. The purposes of this paper are to combine surprisal analysis with other important computation procedures, such as easy manipulation of the analysis results – e.g. to choose desirable result sub-sets for further inspection –, retrieval and comparison with relevant datasets from public databases, and flexible graphical displays for heuristic thinking. The whole set of computation procedures integrated into a single practical tool is what we call *Computational Surprisal Analysis*. This combined kind of analysis should facilitate significantly quantitative understanding of different cellular processes for researchers, including applications in proteomics and metabolomics. Beyond that, our vision is that *Computational Surprisal Analysis* has the potential to reach the status of a routine method of analysis for practitioners. The resolving power of *Computational Surprisal Analysis* is here demonstrated by its application to a variety of cellular cancer process transcription datasets, ours and from the literature. The results provide a compact biological picture of the thermodynamic significance of the leading gene expression phenotypes in every stage of the disease. For each transcript we characterize both its inherent steady state weight, its correlation with the other transcripts and its variation due to the disease. We present a dedicated website to facilitate the analysis for researchers and practitioners.

## Introduction

Surprisal Analysis, in its most general sense, is a procedure to characterize the probability of different states of a system, states that may have a rich internal structure. Furthermore the system may not be in a steady state. The procedure begins by assuming that a set of a relatively small number of constraints is known. These constraints are considered to be sufficient to characterize the deviations of the distribution from the steady state due to the imposed conditions on the system. If the assumed constraints are insufficient to actually reproduce the probability distribution, one is *surprised* and therefore must search for modified and/or additional constraints.

Surprisal Analysis has its basis in the physical sciences and has been successfully applied to a plethora of physical, chemical and engineering problems and convincingly demonstrated to be relevant, useful and producing verifiable results [1]–[4].

The present work belongs to a series of papers [5]–[9] whose purpose is to show that Surprisal Analysis is also relevant and applicable to biological phenomena, in particular cellular cancer processes. A recent commentary on the approach in Biology is [10]. Using surprisal analysis we identify the most stable balanced mRNA distributions at every stage of the disease from the experimental data and also the less stable mRNA networks that maintain the cells away from the balanced state. These networks underlie the process of cancer development. We compare between the cell system/patient networks participating in cancer transformation and relate them to the networks contributing mostly to the balanced state.

This paper has two additional specific purposes.

First, to combine Surprisal Analysis with other important computation procedures, such as easy manipulation of the analysis results – e.g. to choose desirable result sub-sets for further inspection –, retrieval and comparison with relevant data sets from public databases, and flexible graphical displays for heuristic thinking. The whole set of computation procedures integrated into a single practical tool is what we call *Computational Surprisal Analysis*. This combined kind of analysis should be much faster for practitioners and researchers, than having independent but mismatched tools to be integrated into logical and practical consistency.

Second, over a longer time-scale, our vision is to reach the status that *Computational Surprisal Analysis* will be a routine analysis for cancer diagnostics. Thus besides, imaging techniques, minimally invasive surgery, chemotherapy, controlled radiation treatments, it is expected that *Computational Surprisal Analysis* will find its place in clinical practice, speeding-up diagnostics.

Therefore, this paper aims to show:

- the relevance of Surprisal Analysis to the *understanding* of biological phenomena, by discussing novel results in the area of Cellular Cancer Processes in the laboratory environment;
- that *Computational Surprisal Analysis* indeed accelerates Surprisal Analysis, by first describing the integrative aspects of the tool, and then explaining the speed-up gains in computation and in heuristic thinking;
- the applicability of *Computational Surprisal Analysis* to diagnostic of Cellular Cancer Processes, by comparing results obtained for diseased as opposed to healthy subjects.

### Cellular Cancer Processes

Cancer is a highly heterogeneous disease displaying a considerable phenotypic variation among patients with a same type of cancer. Therefore understanding of the underlying oncogenic processes, involved in the process of transformation, requires system-level approaches allowing identification and characterization of the system constituents.

Recent technical advances including cDNA microarrays and mass spec analysis of the cell proteomes, enable to establish global and quantitative functional profiles of cancer cells and tissues. Therefore there is a growing demand for theoretical-computational tools assisting with for the deeper understanding of the data.

Using a theoretical-computational approach we analyzed several gene expression datasets, including renal cancer patients, HPV16 induced transformed keratinocytes and WI-38 transformed fibroblasts [7], [8]. Furthermore the method of analysis can be applied not only to messenger RNAs, mRNAs as we do here but also to microRNAs [9] and beyond to the all –omics datasets, including proteomics and metabolomics.

In this paper we center attention on an analysis of the mRNA levels utilizing the same quantitative principles as in non-equilibrium multicomponent systems in physics and chemistry. Utilizing biological systems evolving in time in response to perturbations we aim to define the mRNA signatures at the most stable, steady state of the system and the groups of mRNAs that deviate from the steady state due to perturbation. For this purpose we utilize surprisal analysis as a technique that enables us to apply thermodynamic principles in biology [4], [6], [8], [14].

The output of surprisal analysis includes several groups of mRNAs, those that contribute mostly to the steady state and other group of mRNAs contributing significantly to the deviations from the steady state at every stage of transformation. The last group comprises highly heterogeneous unstable transcription phenotypes [6] underlying the process of transformation. In addition to identification cancer specific gene/protein signatures, surprisal analysis allows comparing of the disease mRNA phenotypes to the most stable and resistant to perturbations steady state transcription patterns at every stage of the disease, adding a new layer to the characterization of varying parts in the cancer transcriptome.

### Surprisal Analysis

Surprisal Analysis is based upon the principle of maximal entropy. Entropy is a physical quantity that originated in the discipline of Thermodynamics, then appeared in Statistical Mechanics and later on in Information Theory. Qualitatively speaking entropy is a measure of disorder or lack of information. Entropy increases when the chance of a system to be in a given state among its many possible states is more uniform. If the probability of the system to be in a certain state is much larger than the probabilities to be in any other state, we do not lack the information about the system and entropy is minimal.

The approach based upon the principle of maximal entropy, says that our information about a distribution of the system states is obtained by maximizing the entropy under the known information constraints. In absence of any information, the disorder is maximal and the information is minimal.

We impose the constraints using a method introduced by Lagrange (for further details see File S1). It requires maximizing the expression for the Lagrangian  as a function of the Lagrange multipliers:
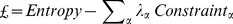
(1)


Each 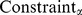 is multiplied by a coefficient 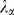 a Lagrange multiplier whose numerical value tells about the relative importance of the respective constraint in the particular circumstance. All the weighted constraints are summed and constrain the Entropy to be reduced from its absolute maximal value.

In our application of this technique to cellular cancer processes, constraints are viewed as so-called transcription/translation patterns/cancer signatures e.g. related to specific cellular processes. These biological patterns prevent cancer cells to reach the maximal entropy that is expected to exist at the balanced state of the biological system. Surprisal analysis identifies both states at every stage of the disease: the balanced state and the constrained state, where specific cancer patterns are most active [6]–[8]. At any given point in time certain patterns contribute more than others. Thereby one can infer about the relative importance of specific cellular processes in different stages of the cancer onset. In this analysis every transcript can participate in more than one transcription pattern, underlying the process of cancer development.

### Computational Surprisal Analysis

**Figure 1:**
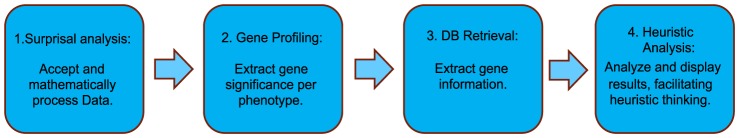
*Computational Surprisal Analysis*. Software Architecture of the integrated tool.

In order to demonstrate the concept and obtained speed-up of *Computational Surprisal Analysis*, an integrated tool was designed and implemented, having the overall software architecture shown in Figure 1. Its software modules (from now on called softmodules) will be described in detail in the Methods section of the paper (for further details see also the File S1).

In order to get the integrative flavor of the *Computational Surprisal Analysis* tool, we now mention the four softmodules' inputs and the final output of the analysis:

1. *Surprisal Analysis* – input is a large rectangular matrix of data of gene expression levels obtained from measurements in a chip array. One of the dimensions of the data matrix is much larger than the other one (for example, 4 time stamps by approximately 22,000 genes). A goal of the surprisal analysis is to reduce the data matrix to manageable dimensions, viz. to obtain a square matrix whose dimension is smaller or at most equal to the small dimension of the data matrix;
2. *Gene Profiling* – input is a small matrix of data whose size is set by the number of patterns relevant to the information measured, say a 4 by 4 matrix, relevant to 4 time stamps in the cellular processes;
3. *DB Retrieval* – input consists of sub-sets of genes obtained by the gene profiling. Each sub-set contains the more influential genes in the respective pattern;
4. *Heuristic Analysis* – input is data obtained in the previous softmodules. Computation is performed to analyze, and interactively display to further analyze heuristically.

**Figure 2:**
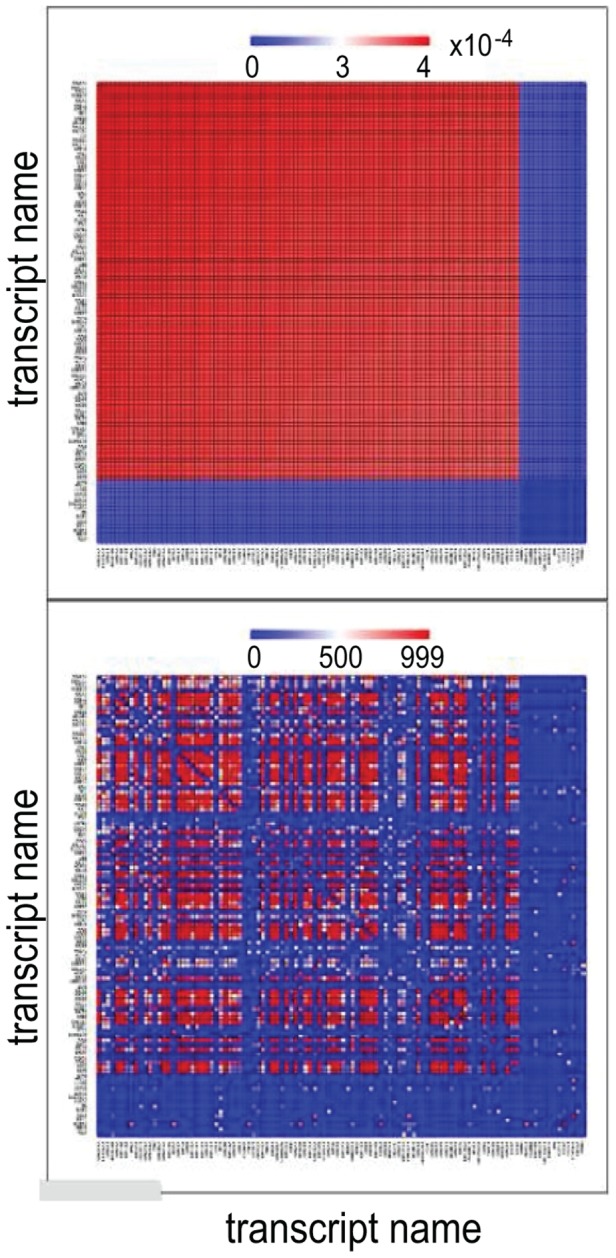
Heat Maps. The upper heat map is obtained by means of Surprisal Analysis. The lower heat map is based upon totally independent data obtained by DB Retrieval. The axes in both heatmaps are identical, viz. they contain the same transcription names in the same order. As usual for heatmaps, colors stand for relative intensities (numerical scales seen above each heatmap): red is high intensity and blue is low intensity. The regions with the same color in both heatmaps clearly overlap, i.e. their results fit very well, implying that *Computational Surprisal Analysis* – in the upper heatmap - can yield predictive information about transcriptional and protein network structures – in the lower heatmap. White dots in the lower heatmap denote lack of information for the specific transcripts.

A sample output is seen in two heat maps, describing protein connectivity, in Figure 2.

## Results

In this section we present results of our work as viewed from three different perspectives: a- genomic characterization of cancer processes: b- the nature of *Computational Surprisal Analysis*; c- the vision of *Computational Surprisal Analysis* as a practical cancer diagnosis tool.

### A- Genomic Characterization of Cancer Processes

For genomic characterization of cancer processes the relevant experimental input are the transcription levels of the different mRNAs. The constraints 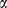 in the surprisal analysis label the phenotypes and a suitable terminology is given by an application and specialization of equation (1) above, as follows:
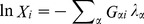
(2)where the indices refer to gene *i* and to the phenotype. 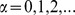 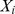 is the experimental expression level of gene *i*, 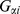 is the (time-independent) extent of participation of a given transcript *i* in the transcription pattern 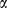 and 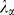, the Lagrange multiplier of equation (1) is here the weight of the respective transcription pattern 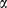. This terminology will be further clarified in the Methods section with particular reference to the special role of the 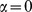 term.

The final output of the *Computational Surprisal Analysis* is the heatmaps showing the extent of participation of the transcripts in particular transcription patterns indexed by 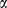. These theoretical heatmaps are compared to the experimental heatmaps describing the functional connectivity of the examined transcripts, using the connectivity scores from the STRING database (See Methods section). In this way we relate 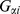 values to the functional networks, having the highest STRING connectivity scores, which were verified experimentally.

The 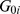 coefficients, where the index 0 refers to the zeroth phenotype, have negative values, meaning that the transcripts most contributing to the *steady state* have the lowest 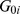 values. 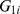 values – for the first phenotype – represent the extent of participation of a particular transcript in the most important transcription pattern underlying the *process of cellular transformation*. 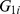 values can be both negative and positive, pointing to the correlation or inverse correlation of the transcripts within the transcription pattern. The transcripts are labeled according to Gene Ontology categories.

#### HF1 cells – HPV16 Immortalized keratinocytes

Using HPV-16 induced immortalized keratinocytes, we analyzed gene expressions between different stages of HPV-16 induced transformation of keratinocytes [11]. Gene expression levels were measured at *four discrete time points*, called respectively:

- K (normal cells untransformed by the papilloma virus),
- E (HPV16 transformed cells from an *early* stage of transformation),
- L (transformed cells from a *late* stage of transformation)
- BP (the cells from the late stage that were treated by *benzo[a]pyrene*) [11].

Using surprisal analysis we identified the major transcription pattern 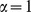 contributing at all time-points (For more details see [8]). This transcription pattern included the transcripts responsible for the shrinkage in the pathways controlling apoptosis and enhancement in the cell cycle networks in the late stages of transformation. All these signatures were validated by biochemical means [11].

Surprisal analysis also identifies secondary transcription patterns that are not significant at all the stages of the HF1 transformation [8]. In this work we examine the most stable transcripts contributing to the balanced, invariant state of the HF1 system and compare them to the major transcription pattern involved in the process of transformation. We use *Computational Surprisal Analysis* to build symmetric matrices – in order to generate heatmaps –, e.g. whose *ij* element is 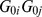.

**Figure 3:**
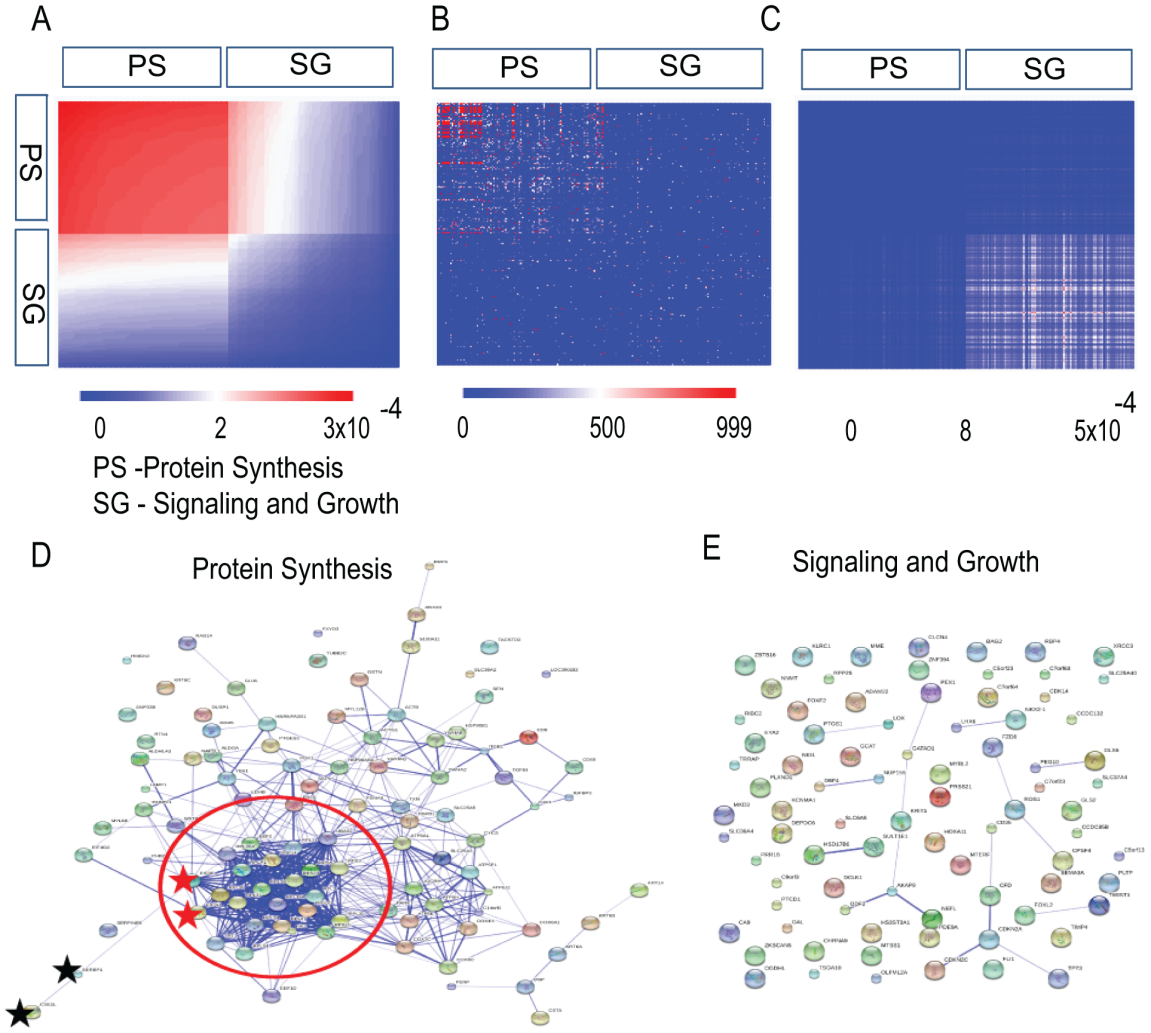
HPV16 Immortalized keratinocytes. (A) A heatmap of the 100 most stable (seen in (a) in red color) and the 100 most contributing to the main transcription pattern 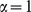 (highest 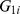) and up-regulated transcripts (seen in (a) in blue color) as obtained by surprisal analysis. In this figure: PS – protein synthesis, SG – Signaling and Growth. (B) A heatmap of the same transcript list in (A) using STRING DB scores. (C) The same transcripts list was utilized to generate 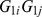. (D) Connectivity Map of the 100 most stable transcripts as using STRING DB; the red color ellipse encloses the most stable and connected transcripts involved in the protein synthesis. The thickness of the lines reflect the approximate probability of the protein-protein functional link for the related transcripts as provided by the String score (see Methods sections for more details). For instance, thick lines (as for the proteins highlighted by red stars inside the ellipse, String score  = 0.999) represent high probability for the functional connectivity based on biochemical verification, whereas thin lines (as for the proteins highlighted by black stars, in the left bottom outside the ellipse, String score  = 0.507) represent smaller probability for the functional connection. (E) Connectivity map of the 100 transcripts most contributing to the main transcription pattern 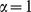 (blue color).

In Figure 3 one can see results for HF1 cells (HPV16 Immortalized keratinocytes) of *Computational Surprisal Analysis* in five different forms. These are respectively:

- Upper left – Heatmap representing 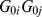 values;
- Upper middle – Heatmap of the same transcripts list in (a) using STRING DB scores;
- Upper right – Heatmap of the same transcripts list in (a) with 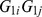 values;
- Lower left – Connectivity Map of the most stable transcripts in (a) using STRING DB;
- Lower right – Connectivity Map of the highest 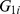.

From Figure 3 one can observe that, the most stable transcripts (with the lowest values of 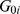 belong mostly to the protein synthesis category. There is a good correspondence between (Fig.3A) and (Fig.3B) heatmaps, meaning that the most stable transcripts, as defined by surprisal analysis, are more functionally connected as shown in the STRING DB heatmap. The heatmap (Fig. 3B) is the quantitative representation of the connectivity maps (Fig. 3D and 3E).

The (Fig. 3C) heatmap of the same gene list with 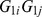 values is uncorrelated with the (Fig. 3A) and (Fig. 3B), meaning that the transcripts with the largest contribution to the stable invariant state hardly participate in the process of transformation. Those transcripts contributing mostly to the process of transformation generate less connected map (Fig.3B, 3E) in comparison with the most stable transcripts (Fig. 3B and 3D) that have very small relative changes (the lowest values of 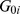).

#### WI-38 cells – WI-38 transformed fibroblasts

**Figure 4:**
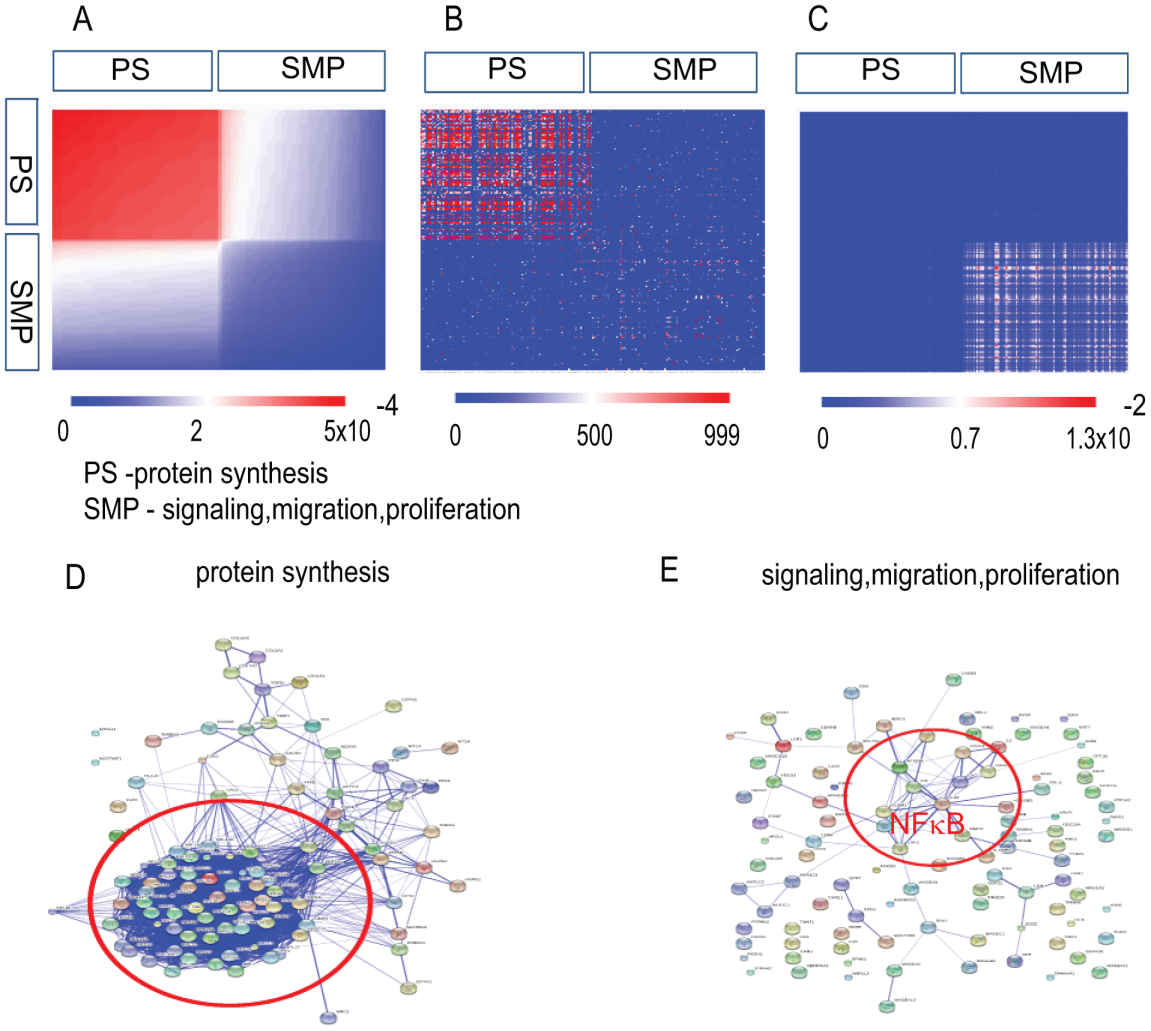
WI-38 transformed fibroblasts. (A) A heatmap of the 100 most stable (seen in (A) in red color) and the 100 most contributing to the main transcription pattern 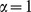 (highest 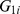) and upregulated transcripts (seen in (A) in blue color) as obtained by surprisal analysis. (PS – protein synthesis, SMP – Signaling, migration, proliferation). (B) A heatmap of the same transcript list in (A) using STRING DB scores. (C) The same transcripts list was utilized to generate 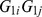. (D) Connectivity Map of the 100 most stable transcripts as using STRING DB; the red color ellipse encloses the most stable and connected transcripts involved in the protein synthesis. (E) Connectivity map of the 100 transcripts most contributing to the transcription pattern 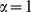 (blue color).

In Figure 4 one can see results for WI-38 cells (WI-38 transformed fibroblasts) of *Computational Surprisal Analysis* in the same five forms and conventions as in Figure 3. This cellular system includes 12 stages of cancer transformation in which different genetic alterations were applied [12]. This cell system underwent about 12 molecular manipulations such as hTERT insertion, cell doublings, repression of p53 function and the insertion of oncogenic H-Ras as reported in [12], thereby developing of the normal WI-38 immortalized non-transformed fibroblasts into fully transformed cells. In this cancer cellular system the balanced state was stable during all 12 time points of transformation, whereas the significance of the transcription patterns involved in the process of transformation varied at different time points [7].

From Figure 4 one makes the same observations as above: there is a good fitness between (Fig. 4A) and (Fig. 4B) heatmaps; the (C) 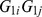 values heatmap is uncorrelated with (Fig. 4A) and (Fig. 4B). The heatmap (Fig. 4B) is the quantitative representation of the connectivity maps (Fig. 4D and Fig. 4E). Transcripts with the highest 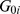 values and the biggest absolute 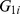 generate less connected maps (Fig. 4E) with several biological modules (not to be confused with softmodules). The main network module in the Figure 4E includes transcripts participating in the NFκB (Nuclear factor kappa B) signaling. Interestingly this module belongs to the additional (minor) transcription pattern 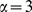 that has large weights in the last stages of cancer development [7]. This module was validated and defined previously as “tumor-forming genetic signature” in the WI-38 cancer model system [12].

One summarizes the Genomic Characterization sub-section by the following points:

1. *Stable networks* (transcripts with the lowest 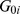 values) generate strong functional connections according to STRING DB. Each protein there is a hub protein, with numerous connections and bridges that can be quantitatively visualized in the surprisal and STRING DB heatmaps. The probability that a lethal mutation, such as deletion, in that hub protein would lead to a cell death is expected to be higher in comparison with the less connected proteins.
2. *Transformation networks and connectivity*– transcripts with the highest 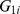 values, contributing mostly to the process of transformation, generate less connected group in all datasets. Thus deletion of one of them or replacement by another protein may not affect significantly the 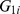 network. The same result was obtained for the transcripts with the lowest 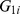 values [6]. As shown above, the 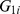 map usually contains several separated networks modules (see for example Fig. 4E). These modules can be further examined as potential targets for the drug therapy.

### B- The Nature of Computational Surprisal Analysis

Here we describe the nature of *Computational Surprisal Analysis*. It essentially consists of the three following aspects: a- synergistic integration of various kinds of computation; b- quantitative speed-up; c- novel kind of inferences exclusively based on surprisal analysis.

#### Synergistic Integration of Diverse Kinds of Computation

Following the softmodules depicted in Figure 1, there are two modes of operation of the *Computational Surprisal Analysis* system:

1- *Sequential* – to concatenate the softmodules exactly as shown in Figure 1, using each softmodule output as the input to the next softmodule.

2- *Cyclical* – certain softmodules are chosen to be cyclically repeated, with possibly varying inputs until one exits the loop, with satisfactory results.

In both ways an efficient computation is essentially limited only by the interactions with the human user. These interactions may be as simple as choosing/reading input/output. They may be more sophisticated, as for example dedicating time to heuristic thinking and making inferences of several types.

In order to enable cyclical repetitions, one must be able to arbitrarily start with a softmodule, independently of other softmodules. This is indeed possible as softmodules are built such that they can either directly receive the output of a previous softmodule in a chain fashion or to get another external input. There is no need to waste time on explicit data manipulation, such as converting formats in between softmodules. This is automatically done, being an intrinsic feature of the synergistic integration.

Concerning the 1^st^ softmodule – Surprisal Analysis – we have already seen that its output includes several groups of e.g. mRNAs: those that participate in the steady state and others that contribute significantly to the deviations from the steady state. The softmodules synergism is necessary to understand the biological meaning of these groups, viz. we utilize e.g. STRING DB access [15] to draw functional networks for every group.

The 2^nd^ softmodule – Gene Profiling – is an efficient integrating bridge between the 1^st^ and 3^rd^ softmodules. It allows selection of the significant genes from surprisal analysis results to retrieve the relevant information from publicly available databases.

Regarding the 3^rd^ softmodule, the access to a database such as STRING DB is done through a suitable interface – transparent to the human user – allowing straightforward selection and retrieval of the desired data into the softmodule, for forward computations. The system modularity enables simple future interfaces to additional databases of interest.

Finally, the 4^th^ softmodule enables relating quantitatively the output of surprisal analysis to the functional connectivity between mRNAs. Two kinds of heatmaps are provided:

1- theoretical heatmap of connectivity using degrees of participations of e.g. mRNAs at the steady state/deviations from the steady state as computed by Surprisal analysis (from the 1^st^ softmodule) and selected by means of Gene Profiling (the 2^nd^ softmodule);

2- functional heatmap calculated from the STRING DB combined scores.

These integrated procedures allow very *efficient and quantitative* understanding of the functional connectivity between mRNAs contributing to the different stages of transformation.

#### Quantitative Speed-Up Evaluation

As seen above, *Computational Surprisal Analysis* involves diverse kinds of computation procedures. These have duration times with very different order of magnitudes, which can be classified as follows:

- *Automatic purely computational procedure* – for instance the Surprisal Analysis performed by the 1^st^ softmodule. The duration of such a computation can be and has actually been measured very precisely. This duration can be certainly shortened by efficient sequential computation in the usual sense or say by parallelization. On the other hand, this is so much faster than the next procedures, that for all evaluation purposes a rough time upper bound of the order of a few seconds is sufficiently satisfactory.
- *Human interactive procedure –* for instance the Gene Profiling of the 2^nd^ softmodule or the slightly longer heatmaps comparison. These are the rate determining steps of the *Computational Surprisal Analysis*. Their duration could be in principle shortened by means of human-computer interaction analysis techniques. On the other hand, it is reasonably safe to assume that its lower bound is limited by human capabilities, roughly estimated to take a time of the order of minutes.

In order to evaluate the quantitative speed-up obtained by the modules of *Computational Surprisal Analysis*, the above duration times should be compared with non-synergistic performance:

- *Manual data conversion and manipulation* – for instance, manually moving the data obtained from databases, while converting them to a suitable format to a heatmap depiction procedure. It could take a roughly estimated duration at least of the order of tens of minutes even for expert software engineers.

From the above estimates, one evaluates the overall quantitative speed-up obtained by *Computational Surprisal Analysis*, to be of the order of ten. This is the ratio between the longest possible duration, viz. the *manual data manipulation* to the shorter rate determining step, viz. the *human interactive procedure* mediated by synergistic automatic data conversion and manipulation.

This faster turnaround enables researchers and practitioners to use the gained time for profitable analysis. In this sense, it speeds-up the potential heuristic thinking. Heuristic thinking has an associative character, as was hinted to by putting side-by-side (for instance in Figures 3 and 4 of the present paper) diverse result displays as Surprisal Analysis generated heatmaps and connectivity maps.

#### An Important Inference: Stability of the steady state

Besides the integration of diverse types of computation with surprisal analysis, the analysis enables new kinds of inferences. Here we discuss the inference of the stability of the basic, housekeeping cellular processes, such as protein synthesis.

The quantitative argument uses eq.(2) that implies that the experimental expression levels of the transcripts with significant (negative) 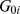 values and small 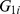 values will be well reproduced using the steady state term only, 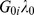. This means that experimental expression levels of these transcripts are not very much influenced by the ongoing deviation processes, as represented by 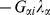 for 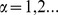, and therefore these transcripts are more stable. By more stable we mean that their expression level may change but only by a fraction, since 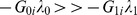.

**Figure 5:**
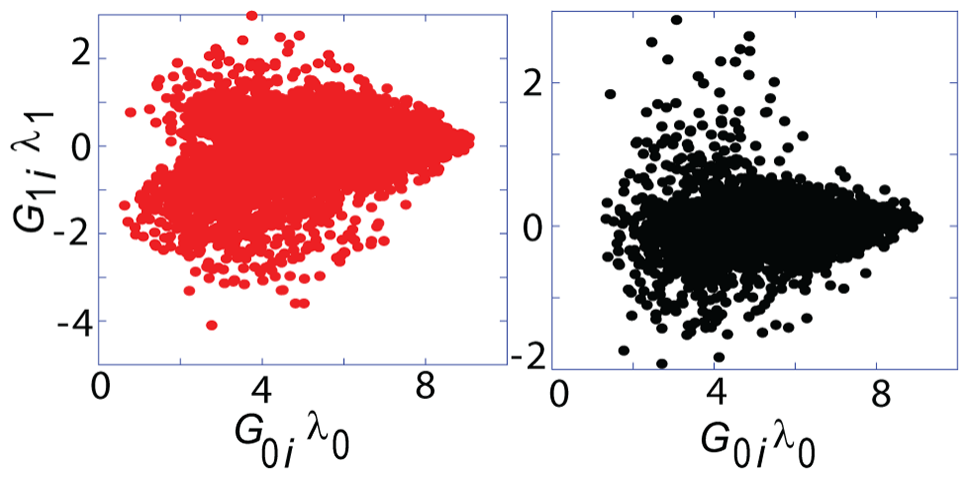
Homeostasis: The stability of the steady state. 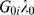
 values for all analyzed transcripts in HF1 cells (left-hand-side red graph) and WI-38 cells (right-hand-side black graph) were plotted against corresponding 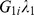 values. Stable transcripts that have small relative alterations contribute less to the process of transformation. The biggest 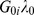 values correspond to low 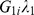 absolute values.

Figure 5 shows HF1 cells and WI-38 cells results from the previous sub-section together, in which 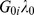 values are plotted against 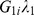 values (representing extent of participation in the carcinogenic process). One can see that the transcripts with the biggest 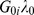 values (those that have lowest values of 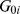) usually have poor participation in the ongoing oncogenic processes (their 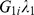 values are close to 0). These transcripts are usually highly expressed in comparison with the less stable and deviating transcripts [6].

Less stable transcripts with significant 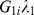 absolute values (transcripts contributing significantly to the deviations from the steady state) correspond to smaller absolute 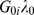 values. In summary, stable transcripts (with the biggest absolute 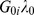 values) have much smaller fold changes and are influenced less by the process of transformation in comparison with the unstable ones (with 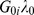 values close to 0 but significant absolute 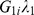 values).

This example of stability of the steady state is interesting as it uses exclusively 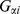 and 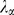 values obtained from surprisal analysis; this kind of inference was motivated by the application of surprisal analysis to cancer characterization.

### C- Computational Surprisal Analysis as a Cancer Diagnostic Tool

What are the advantages of *Computational Surprisal Analysis* as a cancer diagnostic tool? We offer a combination of different reasons. The first refers to types of available information; the second refers to cancer itself as a disease and the third to cancer patients.

Regarding available types of information, recent technical advances enabling quantitative functional profiles of cancer cells and tissues require generation of bio-informatics software tools providing a deeper understanding of the data at the systems level.

Concerning understanding cancer – a very complex disease – working with networks, and not with the individual proteins, is appropriate since many mutations may emerge at the same time due to molecular changes, such as gene mutations and chromosomal instability [16]. Cells that have spontaneous mutations with a survival advantage would proliferate. There is no “golden list” of specific proteins or pathways that provide these advantages. For example, alterations in the “death” network during the transformation is a hallmark of cancer, but different proteins or pathways may lead to that alteration that eventually would result in the same phenotype – cell survival [17], [18]. Surprisal analysis identifies major and minor networks, as represented by transcription patterns, participating in the process of transformation and classifies them according to the importance of every one at each stage of the disease [7], [8].

With respect to patients, cancer is highly heterogeneous, showing a dramatic phenotypic variation between different cancer types and among patients with the same type of cancer [6]. Thus *Computational Surprisal Analysis* has the advantage of enabling fast identification of the patient specific protein/gene signatures along with the characterization of the invariant stable genomic/proteomic reference related to all patients.

As a final example, we present results of the *Computational Surprisal Analysis* of renal cancer patients. We deal with development of renal cancer in three patients [13] and surprisal analysis is carried separately for each patient. We analyze three stages of the disease, namely normal tissues, primary tumor and metastases, and identify the stable balance state in each stage and the deviations thereof. The major transcription pattern accounting for the deviation from the stable state (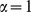) contributed at all stages of the disease in all patients and differentiating between normal and tumor/metastatic tissues.

A list of the most stable (in the balance state) and least stable transcripts (participating in the pattern 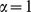) was generated for the patients with renal metastatic cancer. The most stable transcripts (with the lowest values of 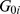) belong mostly to the protein synthesis category and have similar 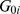 values in all patients.

**Figure 6:**
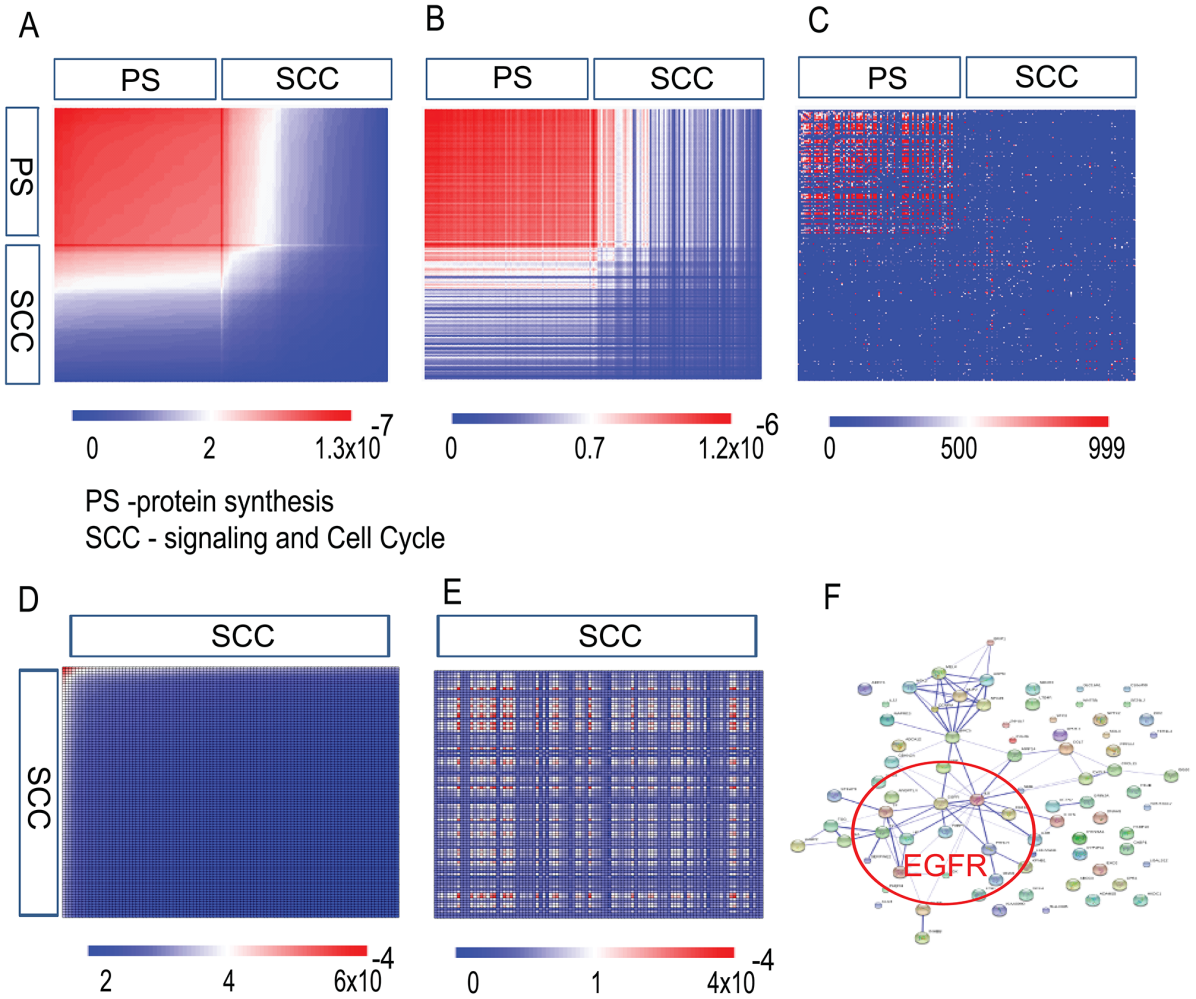
Connectivity of transcripts for Renal Cancer Patients. A list of 200 transcripts was generated for two different cancer patients (A) and (B): a heatmap of 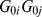 values was obtained using the same list of the transcripts for the two patients. The 100 most stable transcripts (with the lowest values of 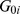) belong mostly to the protein synthesis category and have similar 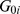 values in both patients. (PS-protein synthesis, SCC –Signaling and Cell Cycle). (C) A heatmap of the same list of the transcripts as in (A) and (B) was generated using STRING DB scores. A good correlation between (A) and (B) and (C) is observed. (D) and (E) Heatmaps of 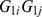 values were obtained using 100 upregulated transcripts with the strongest contribution to the transcription pattern 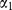 for the two different patients. A bigger heterogeneity was observed among these transcripts. (F) Connectivity map of the 100 transcripts most contributing to the main transcription pattern 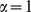 of the patient described in (A) and (D) was generated using STRING DB.

A bigger heterogeneity was observed among less stable transcripts. As was previously mentioned the stable transcripts remained unchanged among the patients, whereas the transcripts participated in the process of transformation varied significantly [6]. Similar results were obtained for two patients with colon carcinoma and for four patients with prostate cancer [6]. Figure 6 shows the relative stability of the protein synthesis network for two of the renal cancer patients.

Although data and respective heatmaps differ in their details, comparison of the patients' heatmaps with that obtained from STRING DB data reveals good correlation. The important point here is that in addition to fast generation of the specific cancer phenotypes for each patient *Computational Surprisal Analysis* identifies a common invariant stable network that remains unchanged between different patients with renal cancer and other types of cancer.

The less stable pattern that strongly contributes to the development of renal cancer differs significantly between examined patients ([6] and Fig. 6D and 6E). This pattern includes proteins participating in the EGFR (Epidermal growth factor receptor) network, such as EGFR and IL6 (interleukin 6), in one patient (Fig. 6F) but not in the other two. EGFR and IL6 are markers of highly invasive tumors, including renal carcinoma [19], [20]. These results point to the potential usefulness of Computational Surprisal Analysis as a candidate patient-oriented cancer diagnostic tool.

## Discussion

We discuss here the results obtained, on-going and future work, and open issues from the three perspectives: a- genomic characterization of cancer processes; b- usage and speed-up due to *Computational Surprisal Analysis*; c- the vision of *Computational Surprisal Analysis* as a potential practical cancer diagnosis tool.

### Genomic Characterization of Cancer Processes

Surprisal analysis identifies a small number of independent transcription patterns that fully describe the process of transformation. At every stage of cancer transformation the importance of every transcription pattern can decrease or increase, thereby giving a very descriptive picture of cancer development process [6], [8]. The most stable transcription pattern remains similar between different cell lines or patients as was shown in this study and earlier [6], [9].

Transcripts that belong to the steady state pattern generate very connected network maps, whereas the transcripts underlying the process of transformation generate much less connected maps with separated small modules. We suggest that a high connectivity of the stable pattern does not allow a big variation between stable patterns of model cell systems or cancer patients in comparison with the unstable and most contributing transcription patterns participating in the process of cancer development.

Using *Computational Surprisal Analysis* the invariant stable transcription pattern along with the unstable patterns are identified. Several small connected modules inside unstable transcription patterns can be usually observed and further examined as drug potential targets, such as the 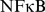 module in the WI-38 cancer module system or EGFR module in the renal cancer patient.

### Usage and Speed-Up due to Computational Surprisal Analysis

Surprisal Analysis is a formal procedure to test a priori hypotheses about complex phenomena. If the hypotheses are reasonable, the same procedure obtains compact descriptions of the relevant probability distributions of the system states, by a few parameters. If the hypotheses are not satisfactory, Surprisal Analysis – as implied by its name – *surprises* us, indicating that the hypotheses must be modified or more parameters added.

In this work we refer to genomic characterization of cancer processes. In these systems the hypotheses being tested can be classified by the following characteristics:

- *Nature and number of intensive variables* – The intensive variables in our systems are the lambda coefficients (the Lagrange multipliers) of the surprisal analysis. As illustrated in the Computational Methods section, the rank of the small matrix used to characterize the cancer process – i.e. the number of phenotypes – is at most the number of the respective intensive variables. Intensive variables determine the kind of comparisons that we wish and can perform.

A typical kind of comparison refers to *time points*. For this case, researchers should decide, based upon a priori biological knowledge, in which time points to perform measurements that are embedded into a chip-array. In such a system, the lambda coefficients, the “potentials”, are time dependent and constitute the relevant intensive variables. For instance, in the HF1 cells – HPV16 Immortalized keratinocytes – *four discrete time points* have been used, therefore a maximum of four phenotypes can be identified.

Another kind of comparison refers to *patients*. If we wish to compare effects on different patients then the relevant lambda coefficients, the “patient potentials”, are patient dependent.

- *Selection and Number of extensive variables* – the common extensive variable in this work is *gene expression*. Researchers use Gene Profiling to select the suitable genes to describe the cancer process behavior for each phenotype.

The *Computational Surprisal Analysis* program has been designed, implemented and made available for *remote open* use for researchers, through the Web. The program offers documentation including a User's Guide and sample input and output, and a reasonable amount of initial support. The program and its documentation are accessible in a Web site (see the Methods section).

As an initial proof of concept, the *Computational Surprisal Analysis* tool has been used by investigators situated in a few locations, in Israel, Europe and the United States. The results reported in this paper were obtained by investigators in two of the mentioned locations. A definitive proof of concept will need much more extensive usage in terms of cancer types, investigator and patient numbers and time period durations.

From a speed-up point of view, *Computational Surprisal Analysis* can identify within several minutes transcription/translation patterns involved in the disease in hundreds and even thousands of cancer patients [9] and assign importance of these patterns to each patient [7]–[9], thereby accelerating the process of patient oriented analysis.

The *Computational Surprisal Analysis* tool has been built with an extensible software architecture and implementation having in mind our main goal to promote fast testing and heuristic thinking in the context of characterization of cancer processes' research. Thus we are open to concrete suggestions, and if necessary even consider partial redesign of the software architecture, while strictly keeping the synergistic integration directives, for additional softmodules such as:

- Complementary relevant algorithms;
- Data selection techniques;
- Access ways to diverse public databases;
- Different kinds of data display.

We are currently working on the development of essential quantitative additions to the *Computational Surprisal Analysis* tool. The following additions will be demonstrated in the next version of the CSA tool:

- new softmodules to make more precise the evaluation of the results obtained, such as: *a- Error estimates* for the results of the tool procedures; *b- Quantitative correlation criteria* for the correlations observed among related heatmaps.
- mobile client in a small dimension generic device, say a smartphone, eventually enabling performance of *Computational Surprisal Analysis* as a diagnosis tool, as discussed next.

Vision: *Computational Surprisal Analysis* as a Potential Cancer Diagnosis Tool

Our vision in the longer term is to enable *Computational Surprisal Analysis* as a cancer diagnosis tool in routine clinical practice (see e.g. [10]). This will demand a few intermediate goals to be achieved.

The first goal is to accumulate results, substantially increasing the confidence in the *Computational Surprisal Analysis* procedures. The results obtained for the renal cancer patients are very preliminary. These are reinforced by similar results obtained for colon and prostate cancer patients [6]. Together, they point out to the desired direction. But extensive use and corroboration of the *Computational Surprisal Analysis* tool is still necessary.

## Conclusion

The main contributions of this work are summarized as follows:

1. *Genomic Characterization* – by contrast with stable gene networks, one can learn about specific groups of genes involved in transformations within cellular cancer processes;
2. *Computational Surprisal Analysis* – a fast and precise approach to genomic characterization. The obtained speed-up enables interactive heuristic thinking for research advancement of cellular cancer processes and opens doors for promising potential diagnostic tools in practice.

## Materials and Methods

### Data sets

Datasets used in the study include HPV-16 induced immortalized keratinocytes [11], WI-38 transformed fibroblasts [12], normal renal, tumor, and metastatic cells from three patients [13].

1. HF1 cells: cDNA was prepared from three independent HF1 cultures each of K, E, L and BP cells and hybridized to the Human Genome U133A Array (Affymetrix) as described [11], GEO accession number: GSE15156.
2. WI-38 System: cDNA was prepared using duplicates from 12 data points. cDNA was hybridized to GeneChip Human Genome Focus Array (Affymetrix) as described [12].
3. Renal carcinoma: cDNA was isolated from three clear renal cell carcinomas including autologous normal tissue and autologous metastasis and hybridized to the HG-U133_Plus2 Affymetrix Human Genome array as described [13], GEO accession number: GSE12606.

### Analysis of mRNA expression data

The gene expression data were analyzed using the Microarray Suite version 5.0 algorithm (Affymetrix). For each probe, a data analysis output file contained:

- a *signal quantitative metric*, which represents the relative level of expression of a transcript;
- a *detection* i.e. a qualitative classification of each signal as present, marginal, or absent;
- a *detection p-value*, indicating the significance of every detection call.

To compare data from different arrays, the signal of each array was scaled to the same target intensity value. For more details see [11]–[13].

After performance of Surprisal analysis the transcripts of interest were divided into biological categories using the DAVID DB [21] and their connectivity was examined by means of retrieved data from the StringDB [15]. We used confidence scores for functional connections that are derived by benchmarking the performance of the predictions against a common reference set of trusted, true associations [15]. The benchmarked confidence scores in StringDB correspond to the probability of finding the linked proteins within the same KEGG pathway [15].

### Computational Methods

The *Computational Surprisal Analysis* program is Web-based, meaning that it can be accessed by a remote client located anywhere [22]. The program was designed and implemented by an object oriented approach [23]. The implementation technology consists of a server running on IIS (Internet Information Services) using C#.net.

Next we provide details (for further details see File S1) about the computation in each of the softmodules (see e.g. [24] for software modularity concepts).

In the 1^st^ softmodule – *Surprisal Analysis* – the main task is to calculate for terms in equation (2), the values of the constraints 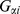 the time-independent extent of participation of a gene transcript *i* in the transcription pattern 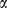 and 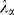 the respective coefficient at time t – a Lagrange multiplier – of 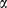 (see e.g. [8]).

**Figure 7:**
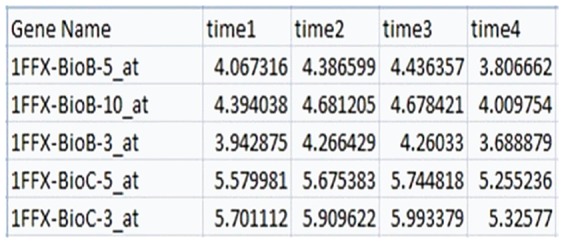
Input file structure sample. It has unique names and expression levels per gene at four time points.

The input, with microarray data uploaded by the user, accepts a CSV (comma separated value) format file, a platform independent standard. All gene names and time names should be unique. Figure 7 shows a partial sample of the input file structure.

The values of 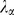 and the constraints 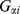 are determined by singular value decomposition (SVD) (see e.g. [25] and references therein; see also e.g. [26] for a different application of SVD to genome data). This procedure extends the notion of matrix diagonalization to rectangular matrices. This is necessary since the number of input genes ***m*** may be very large – say of the order of tens of thousands, while the number of time points ***t***, or another relevant intensive variable of equivalent size, is relatively small – say of the order of ten – thus the input matrix is certainly rectangular.

The output of the SVD procedure consists in two square symmetric matrices whose sizes are quite large – as the number of genes – and quite small – as the number of time points. The rank of these matrices is at most the number of time points. To get the eigenvectors and eigenvalues of these matrices, it is sufficient to solve for the small matrix.

The 1^st^ softmodule output is as follows:

1. *List of genes* – of length *m*, extracted from the input file;

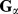
 *vectors* – *t* vectors of length *m*, referred as eigenvectors;
3. *Lagrange multipliers* – a small matrix of size t*t with values of Lagrange multipliers for each time point T and each phenotype 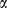.

**Figure 8:**
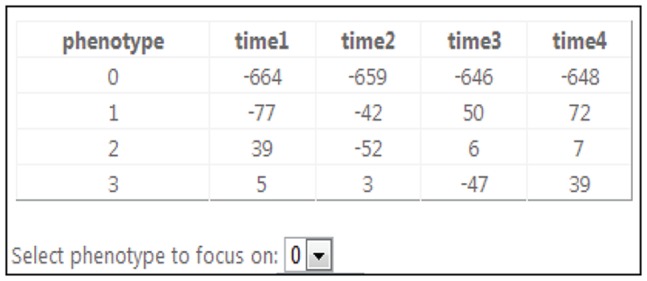
A sample matrix of Lagrange multipliers. Screen print of a particular case showing four phenotypes and four time points.

The small matrix of Lagrange multipliers is illustrated in Figure 8 showing a screen print of the *Computational Surprisal Analysis* tool. This case has 4 phenotypes and four time points. One can also select a phenotype to focus on.

**Figure 9:**
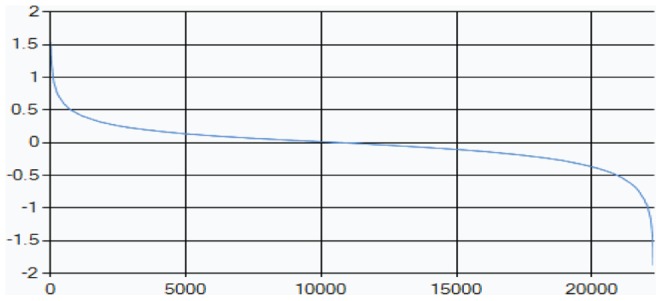
Eigenvector values 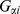 for selected phenotype 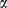. Values are sorted in decreasing order (vertical axis) against running index of genes (horizontal axis).

In the 2^nd^ softmodule – *Gene Profiling* – one interactively selects a sub-set of genes relevant to a certain phenotype. One starts by selecting a phenotype 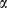 to focus on. Once a phenotype is selected, a graph is displayed in the client screen in which the eigenvector values 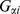 are given sorted in decreasing order (in the vertical axis) for the respective genes ***i*** (running index in the horizontal axis). As seen in Fig. 9, most of the values are around zero, thus not of interest.

**Figure 10:**
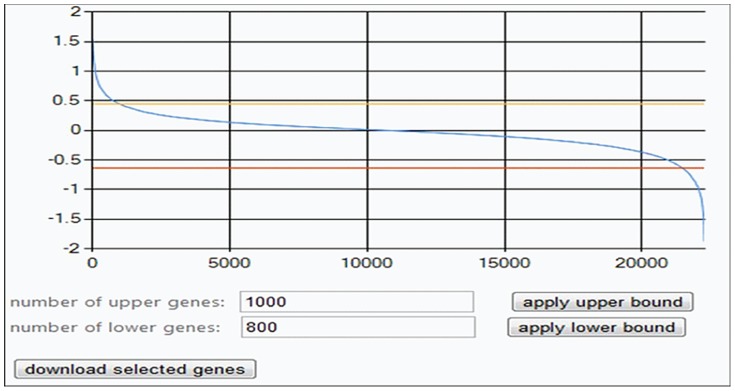
Selected genes with eigenvector values 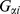 for phenotype 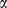. This screen-print shows selected 1000 genes that are above an upper bound (yellow horizontal line) and 800 genes below a lower bound (red line).

The next interactive step is to select smaller sub-sets of genes of interest by applying an upper bound to obtain the desired higher values and a lower bound for the lower values. In the screen print of Figure 10 these bounds are seen as yellow and red horizontal lines. One may then download a list of the selected genes to be used in the next softmodule.

In the 3^rd^ softmodule – *Database Retrieval* – one uses the downloaded list of selected genes to retrieve data from public databases, such as STRING DB [15].

The first task of this softmodule is to enable selection of the desired database. The selection is done based upon a strategy design pattern [27], used to handle communication with different databases. Then it uses the correct unique naming of the relevant genes, making the eventually necessary naming and format conversions.

The 3^rd^ softmodule output for the particular case of STRING DB uses a combined score. For this database various major sources of association data are benchmarked independently. A combined score is computed by STRING DB which indicates higher confidence when more than one type of information supports a given association.

Finally, the 4^th^ softmodule enables infrastructures for heuristic thinking. The infrastructures of this softmodule may be expanded as needed.

**Figure 11:**
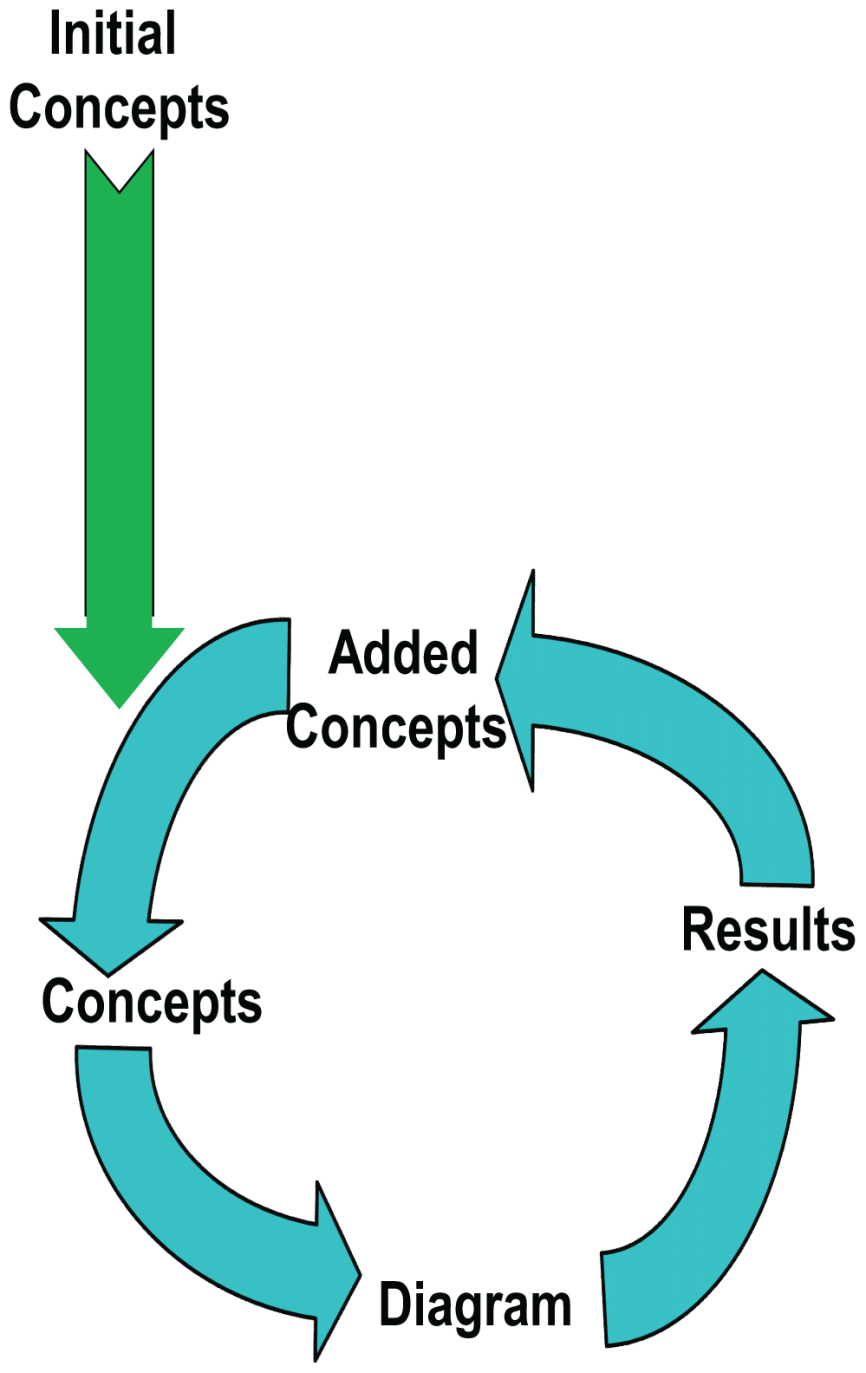
The Heuristic Thinking Cycle. With an initial set of concepts obtained from the surprisal analysis, one performs a computational experiment, whose outcome is a diagram. If one obtains interesting results, one may generalize by inserting this type of diagram in the 4^th^ softmodule and by adding concepts to the subject domain. The cycle may be repeated as many times as desired.

We characterize heuristic thinking in the 4^th^ softmodule to distinguish it from formal deduction. It is experimental, i.e. one performs computational experiments, which are approximate, rather than exact. We envisage heuristic thinking as a cyclic process whose main purpose is to discover new concepts, motivated by original types of visual diagrams. The heuristic cycle is schematically shown in Fig. 11.

The heuristic cycle is illustrated by the process leading to Fig. 2. The *initial concepts* are the 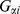 terms from Surprisal Analysis. The *new type of diagram* in this computational experiment is a specific heatmap pair, seen in Fig. 2. The upper heatmap is obtained with the values obtained from Surprisal Analysis. The lower one is obtained from certain values retrieved from StringDB. The *interesting result* is the correlations between heatmaps with corresponding axes with the same transcript names, but totally independent data sources, even spanning different numerical scales. The *new concept* is the predictive power of pairs of 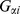 values about transcriptional and protein network structures. *Results are not exact* since data is e.g. lacking in the values retrieved from StringDB.

In our tool a sub-softmodule allows drawing of heatmaps for comparison of Surprisal Analysis results with data retrieved from public databases. Each (non-zoomed in) heatmap has identical labels (genes) in both vertical and horizontal axes.

Specifically, Surprisal Analysis results are computed as products of pairs of the respective 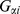 values that “meet” in the specific cell of the heatmap. Heatmaps of STRING DB values are obtained from combined gene connectivity scores.

**Figure 12:**
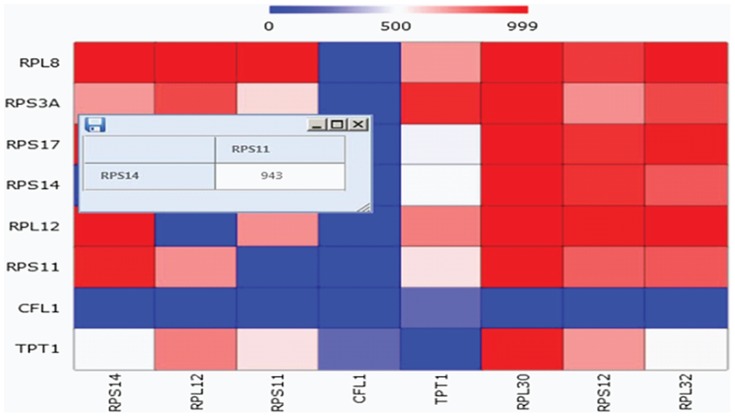
Zooming in on a Heatmap cell. This enables heatmap cell information online – the two crossing genes and the cell value information.

The 4^th^ softmodule output heatmatps that can be zoomed in on online to display heatmap cell information – the two crossing genes and the cell value – for heuristic analysis. This is illustrated in Fig. 12.

## Supporting Information

Figure S1 **CSV file structure sample.** This sample has a title record followed by 5 data records. Each data record has a gene name and numerical expression levels per gene at four time points.
(EPS)

File S1 **Additional detailed information needed for the usage of the Computational Surprisal Analysis program.** This essentially consists of input and output file formats and explanatory material to facilitate understanding of computational features.
(PDF)
