# Supplementary material for: Computational Surprisal Analysis Speeds-Up Genomic Characterization of Cancer Processes
Source: PLoS One. 2014 Nov 18;9(11):e108549. doi: 10.1371/journal.pone.0108549 (PMC4236016; doi:10.1371/journal.pone.0108549)
Supplement: File S1 — Additional detailed information needed for the usage of the Computational Surprisal Analysis program. This essentially consists of input and output file formats and explanatory material to facilitate understanding of computational features. (PDF) [file pone.0108549.s002.pdf]

Supporting Information

Computational Surprisal Analysis
Speeds-Up Genomic Characterization of Cancer Processes

Nataly Kravchenko-Balasha[1], Simcha Simon[2], R. D. Levine[3,4], F. Remacle[5] and Iaakov Exman[2, *]

[1]NanoSystems Biology Cancer Center, Division of Chemistry, Caltech, Pasadena, CA, United States, [2]Software Engineering Department, The Jerusalem College of Engineering – Azrieli, Jerusalem, Israel, [3]The Institute of Chemistry, The Hebrew University, Jerusalem, Israel, [4]Department of Molecular and Medical Pharmacology, David Geffen School of Medicine, UCLA, Los Angeles, CA, United States, [5]Département de Chimie, Université de Liège, Liège, Belgium.

*Email: iaakov@jce.ac.il

Table of Contents

SI1. Computational Material p.2

SI1.1 Overview of the Computational Method p.2

SI1.2 CSV File Format and Manipulation p.5

SI1.3 Lagrange Multipliers and SVD Computation p.6

SI1.4 Gene Profiling p.7

SI1.5 Accessing STRING DB p.7

SI1.6 Heatmap Format and Manipulation p.8

SI2. Glossary p.9

References p.10

SI1. Computational Material

The basic philosophy of the *Computational Surprisal Analysis* program is to treat each of its software modules (in this work coined softmodules) as a black-box that can be smoothly and speedily used, in an integrative fashion, without immersing into the inner workings of each softmodule.

The user with a biological background, in order to speed-up his/her research or eventual diagnostic application, only needs to have a clear notion of inputs, outputs and overall functionality of the softmodules.

The goal-oriented user finds here additional detailed information needed for the usage of the *Computational Surprisal Analysis* program. This information consists essentially of input and output file formats and explanatory material to facilitate understanding of computational features.

Nonetheless, for those readers who wish to delve deeper in the computational background, we also provide further explanations and pointers to the relevant literature.

SI1.1 Overview of the Computational Method

The purpose of this section is to provide the less informed reader with an overview and basic understanding of the computational method, all condensed in a single place. The overview is ordered according to the contents of the four softmodules.

The 1st softmodule – *Surprisal Analysis*

The main purpose of surprisal analysis is to reduce a large amount of data in the input matrix – say gene expression levels measured at different time points – to a much smaller data representation consisting of a small square matrix. The smaller representation's

dimension is given by the “phenotypes” – whose number is e.g. at most the number of time points. In other words, one calculates for terms in the next equation – (see e.g. the supplementary information in ref. [6]):

$$\ln X_i = -\sum_\alpha G_{\alpha i}\lambda_\alpha \qquad \text{(SI-1)}$$

where the indices refer to gene *i* and to the phenotype α. X_i is the experimental expression level of gene *i*, $G_{\alpha i}$ is the (time-independent) extent of participation of a given transcript *i* in the transcription pattern α and λ_α, the Lagrange multiplier, is here the potential (at time t) of the respective transcription pattern α.

The input, with microarray data uploaded by the user, accepts a CSV (comma separated value) format file (see section SI1.2 below). All gene names and time names should be unique. The values of λ_α and the constraints $G_{\alpha i}$ are determined by singular value decomposition (SVD) (see section SI-1.3 below for both Lagrange Multipliers and SVD).

The output of the SVD procedure consists in two square symmetric matrices whose sizes respectively are quite large – as the number of genes – and quite small – as the number ***t*** of time points. The rank of these matrices is at most the number of time points. To get the eigenvectors and eigenvalues of these matrices, it is sufficient to solve for the small matrix.

The 1st softmodule output is as follows:

1. *List of genes* – of length *m*, extracted from the input file;
2. $\mathbf{G}_\alpha$ *vectors* – *t* vectors of length *m*, referred as eigenvectors;
3. *Lagrange multipliers* – a small matrix of size *t*t* with values of Lagrange multipliers for each time point T and each phenotype α

The 2nd softmodule – *Gene Profiling*

The main purpose of gene profiling is to interactively select a sub-set of genes relevant to a certain phenotype. One starts by selecting a phenotype α to focus on. Once a phenotype is selected, a graph is displayed in the client screen in which the eigenvector values $G_{\alpha i}$ are given sorted in decreasing order (in the vertical axis) for the respective genes $\boldsymbol{i}$ (running index in the horizontal axis). Most of the values are around zero, thus not of interest (see section SI-1.4 below).

The interactive step is done by applying an upper bound to obtain the desired higher values and a lower bound for the lower values – effectively rejecting the values around zero. One may then download a list of the selected genes to be used in the next softmodule.

The 3rd softmodule – *Database Retrieval*

The main purpose of database retrieval is to retrieve data from public databases, such as STRING DB (see section SI-1.5 below), in order to allow comparisons of surprisal analysis results with information accumulated from other sources, viz. other kinds of laboratory measurements and inferences.

The first task of this softmodule is to enable selection of the desired database. Then it uses the correct unique naming of the relevant genes, making the eventually necessary naming and format conversions.

The 3rd softmodule output for the particular case of STRING DB uses a combined score. For this database various major sources of association data are benchmarked independently. A combined score is computed by STRING DB which indicates higher confidence when more than one type of information supports a given association.

The 4th softmodule – *Infrastructures for Heuristic Thinking*

The main purpose of the Heuristic Thinking module is to discover new concepts and techniques, motivated by original types of visual diagrams.
It is experimental, i.e. one performs computational experiments, which are approximate, rather than exact.
A typical example in our work is the comparison of a pair of heatmaps, one obtained from surprisal analysis results, the other obtained from retrieved data from public Databases.

SI1.2 CSV File Format and Manipulation

CSV files are used in *Computational Surprisal Analysis* as an input to the Surprisal Analysis softmodule.

CSV means Comma Separated Values (see e.g. RFC 4180 [1] for an informational source with a definition of the CSV format in the context of Internet). This is a simple format in which there are N records, one per line. Each record contains a set of fields, with values separated by a fixed character.

The *Computational Surprisal Analysis* program parses the input CSV file using exactly a comma as the fixed separator character. Note that in principle this could be a different character.

To illustrate the idea, a sample of a CSV file corresponding to Figure 7 in the main-text of the paper is shown in Figure S1.

As a practical consideration, the CSV format is accepted and can be manipulated by the Microsoft Excel application. Thus one can simply convert back and forth Excel xlsx files to CSV files within Excel. In fact one can use and view the contents of a CSV file in the standard Excel display. The sample CSV files provided together with the *Computational Surprisal Analysis* program are thus typically viewed through Excel.

SI1.3 Lagrange Multipliers and SVD Computation

Lagrange multipliers and SVD are computational methods applied in the first softmodule – the Surprisal analysis – of the *Computational Surprisal Analysis* program.

Lagrange multipliers are a mathematical technique to optimize differentiable functions. It is used within Surprisal Analysis to maximize entropy subject to known equality constraints (see e.g. [2]). The Lagrangian, equation (1) of the main text, an expression containing the entropy and the relevant constraints, each multiplied by its respective coefficient – a Lagrange multiplier – is differentiated, obtaining a system of equations that must be solved to obtain numerical values of the coefficients. The numbers λ_α (weights of the biological constraints) in the main text are the undetermined Lagrange multipliers.

Among the many available introductions to the Lagrange Multipliers' technique we choose to point out to the nice tutorial, given by Klein [3] (from the Computer Science at the University of California Berkeley), in order to get the intuition behind its main ideas.

SVD – Singular Value Decomposition – is a mathematical technique [4] used in the first softmodule of the *Computational Surprisal Analysis*, to solve the above mentioned system of equations, represented by matrices. SVD is akin to a solution of an eigenvalue problem for a square matrix. The difference resides in the fact that SVD is necessary because we deal with very rectangular matrices, say with *m* genes and *n* time points, where *m* is much larger than *n*.

The SVD factorization of a real *m*n* matrix ***A*** can be written as:

$$A = U * D * V^T \qquad \text{(SI-2)}$$

where the (left eigenvectors) ***U*** and (right eigenvectors) ***V*** matrices are orthonormal, and the (eigenvalues) ***D*** matrix is diagonal. ***U*** is an *m*m* matrix, ***D*** is an *m*n* matrix and ***V*** is an *n*n* matrix. ***V*** is the small matrix actually solved for the eigenvectors.

It should be emphasized that it is of essential importance that SVD is performed by *Surprisal Analysis* on a matrix ***A*** whose entries are the logarithms of the measured expression levels.

Also for SVD there are available a variety of introductory tutorials. We choose to point out to the MIT BE.400/7.548 course tutorial [5], which uses a gene terminology relevant to the *Computational Surprisal Analysis* application to Genomic characterization (see also e.g. [6] and [7]).

SI1.4 Gene Profiling

Gene profiling – in the second softmodule of the *Computational Surprisal Analysis* program – is done in an interactive way upon a displayed graph of a descending order expression levels against a running index of genes.

The user should choose reasonable sizes of gene sub-sets in the bottom and in the top areas of the displayed graph. To allow later comparison of the generated heatmaps (in sub-section **SI1.5)** these sizes should be fixed across heatmaps.

SI1.5 Accessing STRING DB

Consistently with its basic philosophy, in this initial – proof of concept – version of the *Computational Surprisal Analysis* program, the STRING DB softmodule is accessed in a way totally transparent to the user. It is called as needed within the Heatmap display softmodule.

In future more flexible versions of the *Computational Surprisal Analysis*, which will enable access to different data bases, the user will be asked to provide a minimal amount of information just for the choice of the desired DB.

SI1.6 Heatmap Format and Manipulation

Heatmap charts are a quite common way to display information in a matrix. A heatmap is a discrete chart composed of many small squares, in which each square refers to a (row, column) ordered pair of indices and the square value is represented by a color in a color scale.

The heatmaps generated in the fourth softmodule of the *Computational Surprisal Analysis* display symmetric matrices, in which both the horizontal and vertical axes refer to a chosen sub-set of genes (given by their names). Basically there are two kinds of heatmaps: those computed from the output of the surprisal analysis and those computed from retrieved values from a chosen database. A significant aspect of the heuristic thinking enabled by the *Computational Surprisal Analysis* program is the comparison of the two kinds of heatmaps.

Heatmaps may be zoomed in to facilitate reading of the gene names and the actual numerical value in chosen cells.

SI2 Glossary

The glossary is provided for the benefit of readers with either biological-oriented background or computational-oriented background.

Biological Acronyms and Terms

Affymetrix = a company that manufactures DNA microarrays;

BP = benzo[a]pyrene treated HF1 cells;

cDNA = complementary DNA;

CHP = an Affymetryx file type containing processed information about probe sets;

EGFR = Epidermal growth factor receptor;

HF1 = HPV16 Immortalized keratinocyte human cell line;

HPV16 = Human Papillomavirus type 16;

H-Ras = a Harvey Rat Sarcoma Viral Oncogene Homolog ;

hTERT = human telomerase reverse transcriptase;

IL6 = interleukin 6 encoded by the IL6 gene;

mRNA = messenger Ribonucleic Acid;

NFκB = nuclear factor kappa B;

PS = Protein Synthesis;

p53 = protein 53, a tumor suppressor protein;

SG = Signaling and Growth;

SMP = Signaling, migration, proliferation;

WI-38 = transformed fibroblast human cell line;

Computational Acronyms and Terms

CSA = Computational Surprisal Analysis tool;

CSV = Comma Separated Value, a file format;

DAVID = *Database* for Annotation, Visualization and Integrated Discovery, a database and set of bioinformatics tools;

DB = Data Base;

KEGG = Kyoto Encyclopedia of Genes and Genomes, a database;

Lagrange Multipliers = a mathematical optimization method to find a maximum/minimum of a function subject to equality constraints;

p-value = a statistical significance test;

Softmodules = Software modules within a software system, not to be confused with biological modules;

STRING = Search Tool for the Retrieval of Interacting Genes/Proteins, a biological database, also referred as StringDB;

Surprisal = a mathematical procedure according to the Maximal Entropy approach to obtain a compact description of a distribution of the states of a system;

SVD = Singular Value Decomposition;

References

Note: all the referred web sites below were last accessed in October 2014. See also references in the main-text of the paper.

1. Shafranovich Y (2005) Common Format and MIME Type for Comma-Separated Values (CSV) Files. RFC 4180. Web site: http://tools.ietf.org/html/rfc4180.
2. Gluss D and Weisstein EW (2013) Lagrange Multiplier. From MathWorld --A Wolfram Web Resource. Web site: http://mathworld.wolfram.com/LagrangeMultiplier.html.
3. Klein D (2013) Lagrange Multipliers without Permanent Scarring. Rough draft. Web site: http://www.cs.berkeley.edu/~klein/papers/lagrange-multipliers.pdf.
4. Weisstein EW (2013) Singular Value Decomposition. From *MathWorld*-A Wolfram Web Resource. Web site: http://mathworld.wolfram.com/SingularValueDecomposition.html.
5. BE.400/7.548 Perspectives in Biological Engineering course (2002) Singular Value Decomposition Tutorial. Massachusetts Institute of Technology, Cambridge, MA, USA. Web site: http://web.mit.edu/be.400/www/SVD/Singular_Value_Decomposition.htm.

6. Remacle F, Kravchenko-Balasha N, Levitzki A, Levine RD (2010) Information-theoretic analysis of phenotype changes in early stages of carcinogenesis. Proc Natl Acad Sci U S A 107: 10324-10329.
7. Kuruvilla, FG, Park PJ and Schreiber SL (2002) Vector algebra in the analysis of genome-wide expression data. Genome Biology 2002, 3(3):research0011.1.
